# Effect of Swell-Drying on Mango (*Mangifera indica*) Drying Kinetics

**DOI:** 10.3390/foods11152220

**Published:** 2022-07-26

**Authors:** Luis Alberto Casaverde-Pacherrez, Carmen Téllez-Pérez, Colette Besombes, Daniel Marcelo-Aldana, Karim Allaf, Edilberto Vásquez-Díaz

**Affiliations:** 1 Faculty of Engineering, University of Piura, Piura 20009, Peru; luis.casaverde@alum.udep.edu.pe (L.A.C.-P.); daniel.marcelo@udep.edu.pe (D.M.-A.); 2 Laboratory of Engineering Science for Environment LaSIE-UMR-CNRS 7356, Eco-Intensification of Agro-Industrial Eco-Processes, La Rochelle University, 17042 La Rochelle, France; ctellezperez@gmail.com (C.T.-P.); colette.besombes@univ-lr.fr (C.B.); kallaf@univ-lr.fr (K.A.); 3 Tecnologico de Monterrey, Escuela de Ingeniería y Ciencias, Epigmenio González 500, San Pablo, Querétaro 76130, Mexico

**Keywords:** instant controlled pressure drop (DIC), swell drying, drying kinetics, mango (*Mangifera indica*), starting accessibility, effective diffusivity and apparent drying coefficient

## Abstract

Swell-Drying operation (SD) was applied on mangoes to evaluate its effect on drying kinetics: starting accessibility (*δW*), apparent drying coefficient (*D_app_*), and time to obtain a final moisture content of 20% d.b (t_f_ = 20% d.b). Swell-drying consisted of (1) submitting fresh mangoes to a first pre-drying stage under Convective Air Drying (CAD) until a moisture content of 37% d.b; (2) applying Instant Controlled Pressure Drop (DIC) treatments on pre-dried mangoes by following a central composite rotatable design (steam pressure: 0.2–0.6 MPa and treatment time: 5 and 55 s); and (3) apply post-drying of mangoes under CAD. In both cases, CAD was performed at 60 °C and airflow of 1 m/s. Results showed that both the treatment time and the steam pressure impacted the *D_app_* and the *δW*. By comparing to the control, SD (0.54 MPa and 48 s) increased the *D_app_* and *δW* to 12.2 and 2.7 times, respectively. Moreover, SD triggers a significant reduction in post-drying time (t_f_ = 20% d.b), being this of 2.4 h vs. 30.8 h. These results could be linked to the expansion of the internal pores of mangoes generated by the instant autovaporization of residual water triggered by DIC treatment.

## 1. Introduction

According to the United Nations Conference on Trade and Development (UNCTAD), dried food is becoming a product with great export potential in many markets, indicating that in 2019, the global exports of dried fruit (including dried grapes) amounted to USD 4.13 billion [1]. In 2018, Peruvian dehydrated fruit exports exceeded USD 11 million in FOB values compared to the USD 7.3 million registered in 2017. This means a 49% growth in total dehydrated fruit, with mango and aguaymanto *(Physalis peruviana L*.) being the most exported products. In this respect, mango reached USD 5.9 million in FOB values, with its main export markets being Mexico, the United States, and Germany [2]. This increase in the demand for dehydrated fruits is mainly due to the global trend of consuming healthy and nutritious foods. In addition, during the COVID-19 pandemic, the demand for non-perishable products, especially dried fruit and vegetables witnessed a huge rise in consumer demand [3].

Therefore, to address new consumer preferences, food industries need to look for drying processes that reduce energy consumption and improve the overall quality of final products. Traditional convective hot air-drying operations (CAD) suffer from a poor image in terms of the quality (nutritional, textural, and functional) and in terms of the efficiency of the process (high energy consumption due to long drying kinetics). Due to extensive exposure to high temperatures (in some cases more than 48 h), both nutritional and phytochemicals compounds can be lost [4]. Moreover, due to collapse and shrinkage during CAD drying, generally, these products are hard. In addition, CAD lacks food safety; it allows the presence of microorganisms, especially pathogenic or saprophytic fungi in form of spores [5]. Additionally, in the case of solar drying, the presence of insects, rodents, and birds remains latent throughout the entire process. 

Thus, to improve the quality of CAD dried products, and the efficiency of this drying operation, the “Instant Controlled Pressure-Drop technology”, known by its French acronym DIC (Détente Instantanée Contrôlée), has become a solution to shrinkage/collapse issues [6], and to microbial decontamination [7] and high energy consumption [8]. 

The Instant Controlled Pressure Drop is a thermo-mechanical process that consists of subjecting a product to high saturated steam pressure (around 0.1–1.0 MPa), for a short time (some seconds), followed by an instant pressure drop (ΔP/Δt > 0.5 MPa/s) towards the vacuum (around 5 kPa). The instant pressure drop simultaneously triggers the auto-vaporization of the water (produced as a function of the temperature difference between the initial heating stage and the final equilibrium temperature), the swelling (with a possible rupture of the cell walls), and instantaneous cooling of products, which stops thermal degradation [8]. 

Thus, the coupling of airflow drying to DIC expansion has been defined as “Swell-drying”, and recent studies have shown that this innovative drying process can solve the drawbacks of traditional convective air drying operations [8,9]

Therefore, the objective of this study was to evaluate the effect of swell drying on mango. In this context, this study evaluated the influence of varying the steam pressure and treatment time of DIC texturing and established their influence on drying kinetics, evaluating the starting accessibility, the diffusivity, and the drying time.

## 2. Materials and Methods

### 2.1. Materials

Brazilian mangoes (Kent variety) were bought from a local supermarket in La Rochelle, France. Products were transported to the laboratory and stored for one night at ambient temperature (~20 °C).

### 2.2. Methods

**Figure 1:**
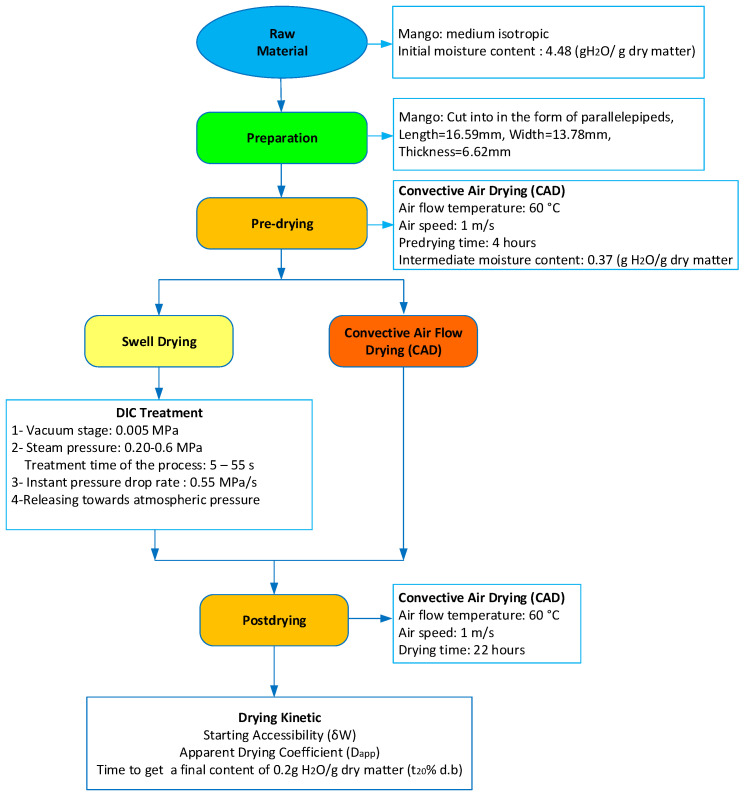
Drying protocol of mangoes.

The drying protocol of mangoes has been described in Figure 1. The pre-drying and the Instant Controlled Pressure Drop experiments were performed at the LaSIE (Laboratory of Engineering Science for the Environment of the University of La Rochelle in France).

#### 2.2.1. Sample Preparation

Before any treatment, mangoes were weighed, washed, peeled, and pitted. After that, they were cut into parallelepipeds with average dimensions of length = 16.59 mm, width = 13.78 mm, and thickness = 6.62 mm. The initial moisture content and the °Brix of raw material were determined.

#### 2.2.2. Moisture Content Determination

Moisture content was determined by following the AOAC 23.003:2003 standard method [10]. For that, 1 g of sample was placed into an oven at 105 °C for 24 h. 

#### 2.2.3. Brix Content Determination

Brix content was determined on mango juice with a digital refractometer (ATAGO SMART-1, France) and expressed as °Brix at 20 °C.

#### 2.2.4. Pre-Drying

Fresh cut mangoes (4 kg) were pre-dried for 4 h into a convective air drier (Memmert D06064UNB 800 Model, Schwabach, Germany) at 60 °C, with an airflow of 1 m/s. The average moisture content of pre-dried mangoes was of 0.37 ± 0.0246 g H_2_O/g dry matter. After that, mangoes were divided into two lots, one for Convective Hot Air Drying (CAD), and the other for Swell-Drying (SD).

#### 2.2.5. Description and Principle of DIC Treatment

Pre-dried mangoes were textured by the instant controlled pressure drop technique (DIC) under different operative parameters of saturated steam pressure (P) and processing time (t), ranging from 0.2 to 0.6 MPa and from 5 to 55 s, respectively. In the section “Experimental design description”, the adopted central composite experiment is described. 

**Figure 2:**
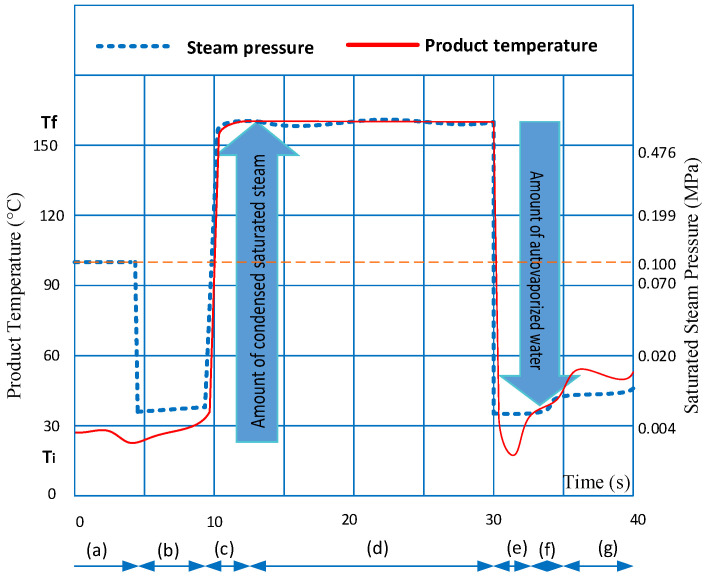
DIC stages applied on mangoes: (**a**) placement of samples into the reactor under atmospheric pressure; (**b**) establishment of initial vacuum; (**c**) injection of saturated steam; (**d**) holding of target saturated steam pressure; (**e**) instant controlled pressure drop towards the vacuum; (**f**) holding of vacuum; (**g**) release of samples at atmospheric pressure.

In this study, the DIC process consisted of seven steps. Figure 2 shows the different steps of applied DIC treatment. First, 250 g of pre-dried mangoes was placed into the reactor (Figure 2a), and to ensure rapid contact between the saturated steam and the sample, a vacuum was created (3–5 kPa) (Figure 2b). Secondly, saturated steam was injected into the reactor until a fixed saturated steam pressure level was reached (Figure 2c), and the target steam pressure was held for some seconds (Figure 2d). Thus, by applying a central composite rotatable experimental design, this stage seeks the homogenization of the temperature and the water into the product; heat transfer is carried out mainly by steam condensation. Thirdly, once the temperature and water content levels were almost homogenized in the material, samples were submitted to an instant controlled pressure drop (0.55 MPas^−1^) towards the vacuum (3–5 kPa) (Figure 2e). At this stage, the autovaporization of water inside the product generates a quantity of steam and a significant mechanical tension that allows the expansion of the product. In addition, the autovaporization of the water guarantees rapid cooling, which prevents the thermal degradation of sensitive compounds and, therefore, guarantees the high quality of the treated products. Finally, after some seconds under vacuum (Figure 2f), to recover the product from the reactor, the ambient air was introduced to return to atmospheric pressure (Figure 2g). 

**Figure 3:**
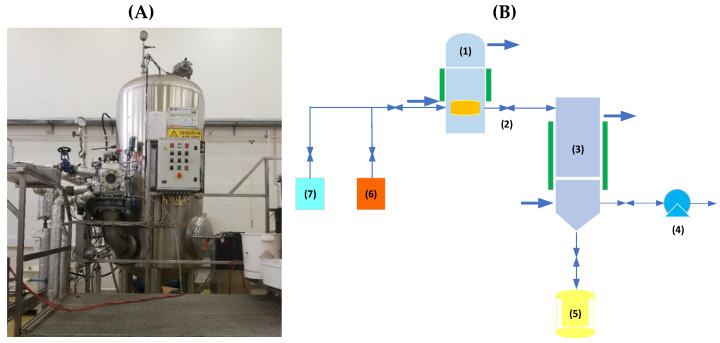
(**A**) Picture of the DIC MP reactor. (**B**) Schematic representation of a typical DIC reactor: (1) treatment vessel, (2) instant controlled pressure drop valve, (3) vacuum tank with cooling jacket, (4) vacuum pump, (5) extract collection trap, (6) steam generator, and (7) air compressor.

For this study, the DIC MP reactor (manufactured by ABCAR-DIC Process; La Rochelle) was used. Figure 3 shows the picture of the DIC MP reactor (A) and its schematic diagram of the reactor (B).

#### 2.2.6. Drying Kinetics at the Post-Drying Stage

After DIC treatment, drying kinetics of the post-drying stage was carried out. For this, 20 g of mango of each experiment (DIC and CAD) was dried under the same conditions and equipment of the pre-drying stage. Weight changes were measured at 1, 5, 10, 15, 30, 60, 90, 120, 180, 240, 300, 360 and 1320 min. Samples’ weights were recorded (using an electronic balance PS 2500.3Y model Radwag, Poland). Equilibrium water content was determined until the sample’s weight changed to less than 0.01 g for 2 h. 

#### 2.2.7. Mathematical Modeling of Drying Kinetics

Dehydration involves the simultaneous application of heat and the removal of moisture from food. Then, to study the dehydration kinetics of CAD and SD mangoes, the study of Mounir and Allaf (2009) [11] was adopted. This study focuses on the four heat and mass transfers that occurred during drying:

(1) Heat transfer from the outside to the surface of the product; the energy can be supplied by conduction, convection, or radiation.

(2) Heat transfer within the product; the energy is transmitted by conduction.

(3) Water transfer within the product, which takes place in the liquid phase by various processes including capillarity and molecular diffusivity (the driving force is the water content gradient); and/or vapor (the driving force is the vapor partial pressure gradient).

(4) Vapor transport from the surface to the outside.

Then, when the external heat and mass transfers do not limit the whole operation through adequate airflow temperature, relative humidity, and velocity, only internal transfers may intervene as limiting processes. In such conditions, the Mounir and Allaf (2009) [11] model proposed a Fick-type relation:

(1) $$\frac{\rho_{w}}{\rho_{d}}\left({\vec{\nu }}_{w}-{\vec{\nu }}_{d}\right)=-D_{eff}\vec{\nabla }\left(\frac{\rho_{w}}{\rho_{d}}\right)$$

By assuming any structure modification ($\rho_{d}$ = constant and $\nu_{d}$ = 0), the hypothesis of both structural and thermal homogeneities and a one-dimensional flow, the whole drying process is only controlled by mass transfer:

(2) $$\frac{\partial \rho_{W}}{\partial t}=D_{eff}\frac{\partial^{2}\rho_{w}}{\partial x^{2}}$$

Then, in this study Crank’s solution was adopted [12]:

(3) $$\frac{W-W_{\infty }}{W_{0}-W_{\infty }}=\overset{\infty }{\underset{i=1}{\sum }}A_{i}exp\left(-q_{i}^{2}t\right)$$

where *W*_1_ is the value of W at the time t_1_ chosen as the beginning of the diffusion model obtained only for long-time experiments. Coefficients of Crank solutions Ai and qi are given according to the matrix geometry Fick’s number (*τ*) defined as:

(4) $$\tau =D_{eff}*t/d_{p}^{2}\;$$

where *d_p_* is the characteristic length (m), calculated as the relation between the volume and the surface of the parallelepipeds (d_p_ = V/S = a * b* c/2 (ab + ac + bc). For this case, an infinite plate is considered. Then, by limiting Equation (3) to its first term, it could be expressed as:

(5) $$\frac{W-W_{\infty }}{W_{0}-W_{\infty }}=\mathrm{Aexp}(-\mathrm{kt})$$

The logarithmic representation of Equation (5) as a straight line leads to determining *D_eff_* from the slope *k*:

(6) $$ln\left(Y\right)=ln\left(\frac{W-W_{\infty }}{W_{0}-W_{\infty }}\right)=kt\;$$

where *k* corresponds to:

(7) $$k=\frac{\pi^{2}D_{eff}}{4d_{p}{}^{2}}\;$$

and the effective diffusivity is:

(8) $$D_{eff}=\frac{4d_{p}{}^{2}}{\pi^{2}}k$$

Moreover, because the transfer mechanisms during drying are much more complex than diffusion, this model was verified experimentally by plotting the straight line given by equation 6 by excluding the points close to *t* = 0. However, when the diffusion process does not control the drying operation, it cannot be possible to determine the effective diffusivity from the experimental data. In fact, if the kinetics of airflow drying depends on the velocity, it means that the water diffusion within the material is not the limiting process (Absence of Negligible External Resistance); thus, to verify that external heat and mass transfers are not limiting, the Critical Airflow Velocity (CAV) equation from [13], could be used.

Because, in this study, we only evaluated one condition of air flow (1 m/s), we cannot calculate the CAV value, and therefore, we cannot guarantee that external heat and mass transfers are not limiting. Then, in this study, the obtained results from Equation (8) can be considered as apparent drying coefficient (*D_app_*), which becomes a useful tool to compare drying performance between the different drying treatments.

On the other hand, to evaluate the quantity of water dried from the surface regardless of the diffusion process, the “starting accessibility” was determined (δW). Then, through the extrapolation of the diffusional model, the moisture content at *t* = 0 (*W_0_*) can be determined, which is generally different from the initial moisture content *W_i_*. Thus, the starting accessibility was determined as $\delta W=W_{i}-W_{0}$.

Finally, to determine the effect of the DIC treatment on the drying time, Equation (6) was used to calculate the time in minutes for obtaining a final moisture content of 20% d.b from an initial moisture content of 30% d.b (t_d20%_). 

#### 2.2.8. Experimental Design

**Figure 4:**
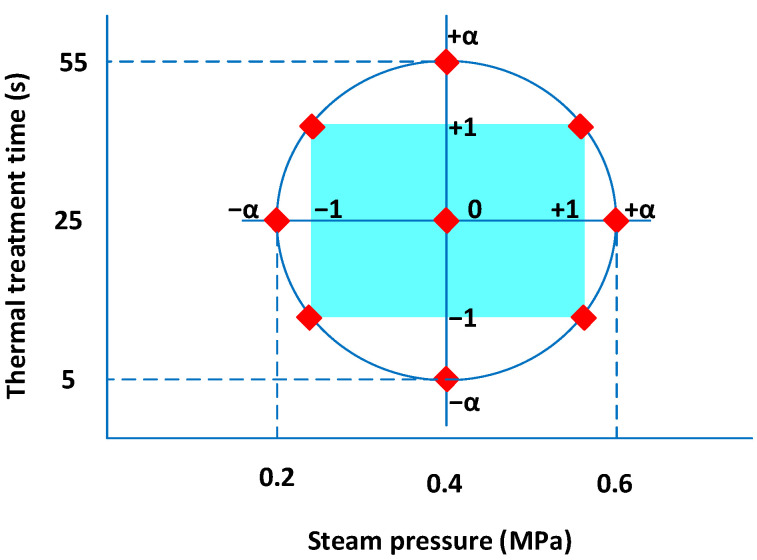
Five-level central rotatable experimental design, with steam pressure “P” and heat treatment time “t” as DIC operating parameters.

A five-level central composite rotatable experimental design was used to assess the effect of DIC operating parameters on drying kinetics. After preliminary tests, the saturated vapor pressure “P” (MPa), with a range between 0.2 and 0.6 MPa, and the treatment time of the process “t” (s) between 5 and 55 s, were used as independent variables (*n* = 2). Thus, the studied design included 11 experiments: 4 factorial points ($2^{n}=2^{2}=(-1/-1;-1/+1$;+1/−1, and +1/+1); 4 start trials (2∗n)=2x2=(−α/0;+α/0;0/−α and 0/+α); and 3 central points (0,0). The value of α (axial distance) was determined as a function of the number (*n*) of operating parameters, and it was calculated as $\sqrt[{4}]{2^{n}}=1.41424$. Figure 4 shows the selected independent variables at five levels of (−α, −1, 0, +1, +α), and Table 1 shows the applied experimental design.

To minimize the effects of unexpected variability in the observed responses due to exogenous factors, the DIC treatments were performed randomly.

#### 2.2.9. Statistical Analysis

To determine the influence of the DIC process variables (steam pressure and treatment time) on (1) the apparent drying coefficient (*D_app_*), (2) the starting accessibility (δW), and (3) the time for obtaining a final moisture content of 20% d.b (t_d20%_), the analysis of variance (ANOVAs) with a *p* ≤ 0.05 was performed to determine significant differences between independent variables. The ANOVA tests was used to evaluate the statistical significance of each of the effects by comparing the root mean square to an estimate of the experimental error. In this case, probability values less than 0.05, indicates that the evaluated variable is significant at 95.0% confidence level. A Pareto chart was used to identify the impact of DIC variables on responses. To optimize the responses, surface response plots were used. In the case that the R^2^ of fitting models had enough accurate to real data, empirical models were presented. The Statgraphics^®^ Centurion 16 software was used to analyze all data.

## 3. Results

### 3.1. Raw Material Characterization

After peeling and pitting, the percentage of high-quality mango pulp was 55%, and peels, stones, and pulpier waste represented 45%. Moreover, the initial moisture content of pulp was 81.75% on a wet basis or 4.48 g H_2_O/g solids on a dry basis. Finally, the pulp °Brix was 18.61. This result agrees with the study of Gurumeenakshi et al. [14], who describe that depending on the variety, mangoes compositions vary between 45 and 65% of pulp, 15 and 20% of peel, 15 and 20% of pulpier waste, and 10 and 20% of stones. On the other hand, according to Dick et al. [15], the soluble dry extract after mangoes ripening varied from 14.2 to 20 °Brix, agreeing with our result. Thus, knowing the ripening stage of fruit could be worthy to ensure the best quality of dried mangoes.

### 3.2. Drying Kinetics at the Post-Drying Stage

**Figure 5:**
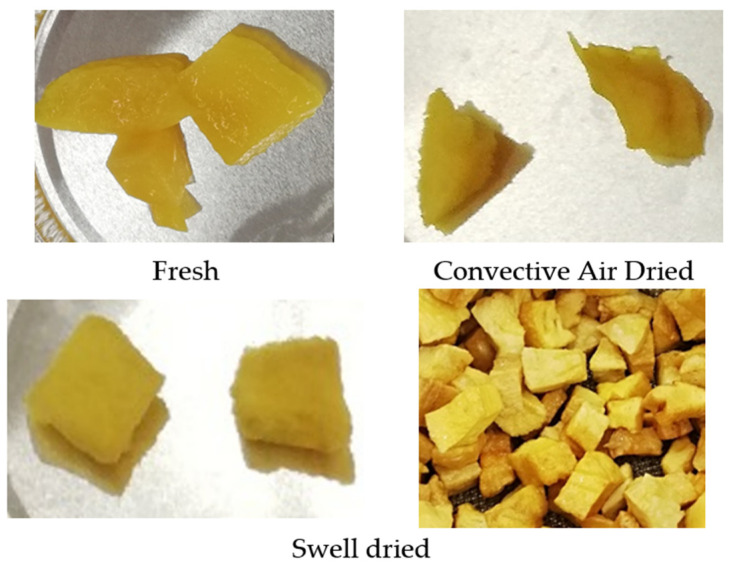
Photographs of Fresh, Convective Air-Dried, and Swell-Dried mangoes.

**Figure 6:**
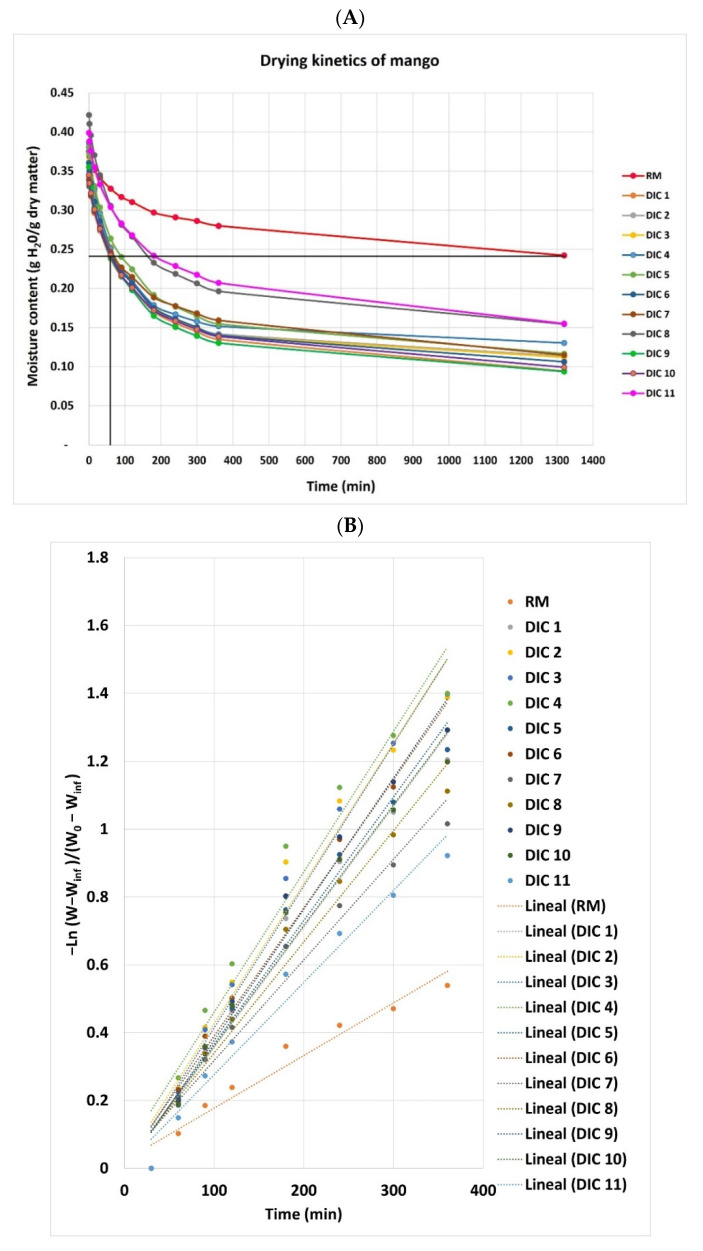
(**A**) Drying curves of raw material (RM) and swell-dried mangoes (DIC) and (**B**)–ln(Y) vs. time (min) plot.

The drying kinetics were studied from an initial moisture content of pre-dried mangoes of an average of 0.37 ± 0.02 g H_2_O/g dry matter. Figure 5 shows the appearance of Fresh, Convective Air-Dried, and Swell-Dried mangoes. Table 2 shows the evolution of moisture content (d.b) in the function of the time *W = f(t)* obtained from experimental drying kinetics, and Figure 6 shows the drying curves of raw material (RM) and swell-dried mangoes (DIC). As observed in the drying curves from Figure 6A, to achieve a final moisture content of 0.24 g H_2_O/g dry matter, RM required 22 h. On the contrary, all swell-dried samples required less than 3 h; for DIC 1, DIC 6, DIC 9 (0.40 MPa and 30 s), and DIC 10 (0.20 Mpa and 30 s), the time was reduced to only 1 h. On the other hand, after 22 h of drying, all swell-dried samples performed final moisture contents lower than 0.16 g H_2_O/g dry matter, both DIC 1 and DIC 9 were the samples that performed the lowest final moisture content (0.09 g H_2_O/g dry matter). According to the United Nations [16], free-preservative-dried mangoes shall have a moisture content not exceeding 15% on a wet basis or 0.17 g H_2_O/g dry matter on a dry basis. In addition, in the case of mangoes treated with preservatives, moisture content should not exceed 25% on a wet basis or 0.33 g H_2_O/g dry matter on a dry basis. Then, regarding Table 2, DIC samples 1, 2, 9, and 10 can achieve in only 3 h of post-drying, a safety level of moisture content, while RM will require the use of preservatives. This indicates that the DIC treatment significantly and systematically reduced the drying time.

To better evaluate the impact of the DIC treatment on the drying kinetics of mangoes, (1) the apparent drying coefficient $D_{app}$ of the water within the matrix, (2) the starting accessibility ratio δW, and (3) the drying time necessary to reach the final moisture of 0.2 g H_2_O/g dry matter from 0.3 g H_2_O/g dry matter initial moisture content were calculated (t_f_ = 20% d.b).

#### 3.2.1. Apparent Drying Coefficient 

**Figure 7:**
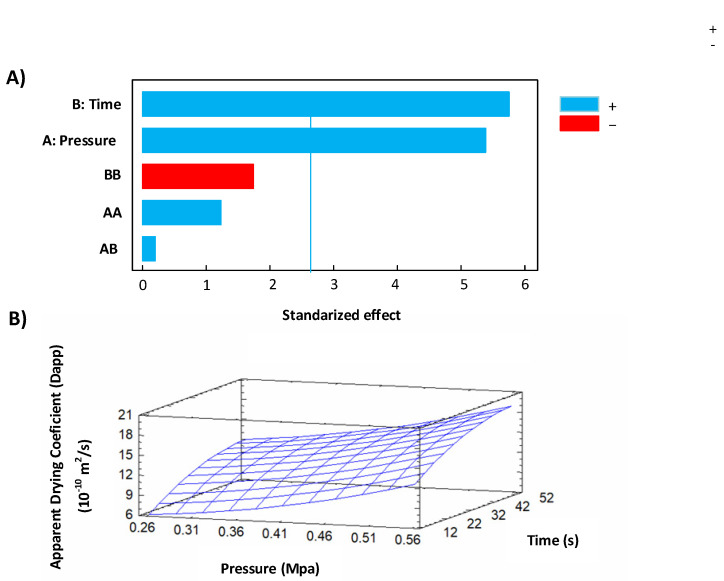
Effect of DIC treatment on effective diffusivity: (**A**) Pareto chart and (**B**) response surface graph.

Table 3 shows the experimental and the fitting model results of *D_app_* for controls and swell-dried mangoes. Concerning the apparent drying coefficient, RM presented an average measure of 1.55 × 10^−10^ m^2^/s, and swell-dried samples performed values between 4.78 and 18.92 × 10^−10^ m^2^/s. The lowest *D_app_* was obtained under DIC 11 (0.2 MPa and 30 s), and the highest values under DIC 4 (0.54 MPa and 48 s). Figure 7A shows that under the selected range of operating parameters, both the saturated steam pressure and the time had a significant effect on the *D_app_* response, and the response surface graph (Figure 7B) shows that the *D_app_* could be increased under high steam pressure values and high treatment times. Figure 7B also displays the fitting model for this response. By using the fitting model, a steam pressure of 0.59 MPa and a thermal treatment time of 55 s will be the optimal conditions to increase the *D_app_* to values near 20.50 × 10^−10^ m^2^/s.

(9) $$D_{app}=3.02835-14.9036*P+0.362541t+43.3356P^{2}+0.0402183P*t-0.00342908t^{2}$$

The ANOVA for D_app_ is shown in Table 4.

#### 3.2.2. Starting Accessibility

**Figure 8:**
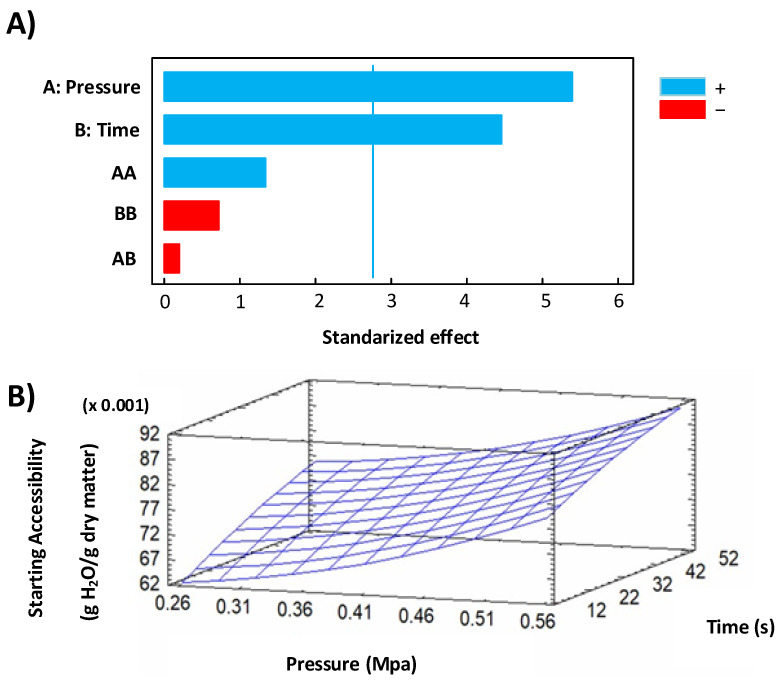
Effect of DIC treatment on starting accessibility: (**A**) Pareto chart and (**B**) response surface graph.

During the short-time first drying stage, vapor transport occurs by sweeping away a vapor rate depending on: (a) the airflow velocity through the evolution of the mass convection coefficient, (b) the vapor density, and (c) the specific surface vapor pressure at the material surface—issued from the airflow temperature, the water activity, the airflow humidity, and the total pressure [17]. Then, in this study, the starting accessibility (*δW*) was used to determine the quantity of water removed at this first stage of drying, and the obtained results showed that DIC treatment systematically increased this response by performing values between 0.060 and 0.090 g H_2_O/g dry matter, vs. RM, which presented values of 0.03 g H_2_O/g dry matter. In addition, under DIC 4 (0.54 MPa and 48 s), the *δW* was increased by 2.7 times. Table 5 shows the *δW* for RM and swell-dried mangoes. Regarding the Pareto chart in Figure 8A and the response surface graph in Figure 8B, it can be noted that under high steam pressure values and high treatment times, the starting accessibility could be increased. Moreover, the equation of the fitted model allowed us to determine the optimal DIC treatment conditions to maximize the *δW* to values of 0.094; with a steam pressure of 0.59 MPa and a thermal treatment time of 55 s. Then, both parameters established the suitability of DIC as a pretreatment stage for higher performance of hot air drying.

(10) $$\delta W=0.059005-0.0407456*P+0.000554176*t+0.120217P^{2}-0.000188492P*t-0.00000244983t^{2}$$

The ANOVA table of Starting_accessibility is shown in Table 6:

#### 3.2.3. Time for Obtaining a Final Moisture Content of 20% d.b

**Figure 9:**
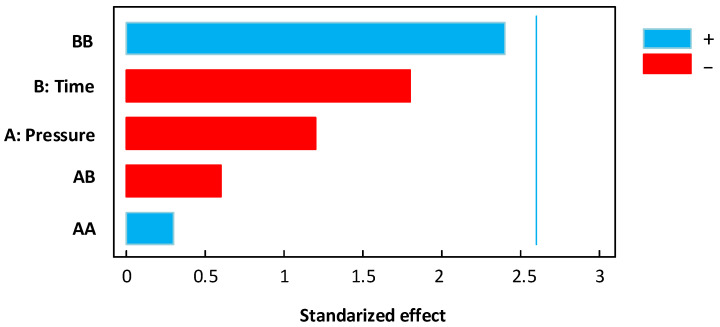
Pareto chart: Effect of DIC treatment on the time for obtaining a final moisture content of 20% d.b.

To evaluate the whole impact of DIC treatment on the drying time of mangoes, in this study, it was calculated the total time needed to achieve a final moisture content of 20% d.b. Then, as can be observed in Table 7, DIC 9 treatment drastically reduced the drying time to 12.6 times concerning the RM (144 min vs. 1849 min). In addition, all the DIC treatments reduced the drying time to between 144 and 314 min. Regarding Figure 9, under the selected operative parameters, this response cannot be accurately explained by the steam pressure and thermal treatment time.

(11) $$Time\;of\;getting\;20\%=339.314-160.753*P-6.7897*t+226.031*P^{2}-4.89858*P*t+0.122754*t^{2}$$

## 4. Discussion

Food drying operation has numerous key issues in process performance (e.g., long kinetics, which implies high energy consumption) and in product quality (organoleptic and nutritional losses due to the long drying periods); moreover, in cases of solar or sun drying, there are high risks of microbial contamination. Therefore, to effectively intensify food drying processes, it is necessary to identify the main operation stages and their limiting process. In the case of convective air flow drying (CAD), there are three different main stages: (1) the first stage of purely superficial evaporation and sweeping away the vapor; (2) the second stage of internal diffusional liquid water transfer within the porous material, and vapor generation and transport from the exchange surface towards the external surrounding medium; and (3) the paradoxical stage of drying generated by a higher vapor pressure at the surface than in the core [18]. Thus, each stage can be addressed with an intensification strategy. 

In the first stage, the intensification can take place by reducing the airflow humidity, increasing the airflow velocity up to critical airflow velocity (CAV), and by rising the airflow temperature. When these parameters have been adjusted, the drying operation becomes negligible external resistance (NER). In the second stage, under NER conditions, the internal diffusion becomes the most significant transfer resistance and the limiting process; therefore, to intensify this stage, it is necessary to increase the effective exchange surface area and to increase the porosity of the food material [19]. Thus, at this stage, the Instant Controlled Pressure Drop (DIC) has allowed a well-controlled modification of the product texture, addressing the shrinkage and collapse, which are the most awkward and key problematic aspects of CAD of food.

In this study, DIC treatment impacted both the first and second stages of drying. In the first stage, regarding the starting accessibility results, DIC removed 2.7 times more water from the surface than RM. This result can be linked to the autovaporization of water after the instant pressure drop stage of DIC treatment, and also to an increase in the exchange surface due to texturing. On the other hand, in the second stage, results showed that DIC increased the apparent drying coefficient (*D_app_*) under all selected studied parameters; with DIC 4 (0.54 MPa and 48 s) being the treatment where the *D_app_* was increased 12.2 times. This result could be linked to the expansion of the internal pores of mangoes generated by the instant autovaporization of residual water after the pre-drying stage, which remedies collapse and shrinkage. 

Thus, DIC treatment improves both the starting accessibility and the apparent drying coefficient, which triggers a significant reduction in post-drying time. To achieve a final moisture content of 0.24 g H_2_O/g dry matter, DIC 1, 6, 9, and 10 required only 1 h vs. 22 h for RM. Thus, a reduction in the drying time can be translated into energy savings and quality improvement. In previous studies, swell drying processing has prevented the overheating of products and the loss of flavor and nutritional values due to long drying times; in addition, the bioavailability of phytocompounds, such as antioxidants, has been increased by DIC texturing treatments, being equal to or higher than freeze-dried products [20,21]. Moreover, the crisp texture of swell-dried foods provides a better sensorial attribute for consumers [6]. 

On the other hand, concerning the third stage of drying (the paradoxical stage), this study did not apply an intensification process; however, future studies could evaluate the use of microwave drying, superheated steam drying, or multi-flash drying to generate high-pressure vapor within the pores, and a possible efficient permeation transfer (Darcy’s permeation transfer) [17]. 

Moreover, it can be added that interval drying, or intermittent drying can also improve the drying operation by favoring the energy use for sweeping water from the surface only when it is necessary, and not during all drying operation. A very similar approach was taken by the intermittent drying of mango study of [22], who implemented the Reaction Engineering Approach (REA). In their study, it has been recognized that heat is need for water evaporation, and that by applying a time-varying temperature profile it can be resulted in a reduction in the effective drying time. 

Finally, it can be highlighted that the swell-drying process can be performed by coupling solar or sun pre-drying operations. DIC provides strong decontamination by eliminating vegetative microorganisms and spores; thus, DIC can also be applied as a decontamination process in drying operations [7,23]. 

Regarding diffusion [24], an analysis is made where it is stated that to generate the diffusivity function, several sets of experimental data must be extracted. Furthermore, it is necessary to perform complex optimization procedures afterward, to represent the dependence of diffusivity for moisture content and/or temperature.

On the other hand, in this same work, it is mentioned that the REA is an application of chemical reactor engineering principles to model the drying kinetics on which evaporation is modeled as first-order kinetics with activation energy, while condensation is modeled as zero-order kinetics with no activation energy. The DIC also considers these processes as important stages, to achieve surface drying at the beginning where condensation is manifested, and self-evaporation in the stage of abrupt pressure drop. Therefore, REA is also an alternative for modeling diffusion-based drying processes.

## 5. Conclusions

Mango is one of the most consumed tropical fruits in the world; however, it is highly perishable. Therefore, to increase its shelf life, convective air drying (CAD) is widely applied as a preservation method, even though it suffers from a poor image in terms of both quality and efficiency of the process.

Drying aims to remove the water present in the product through three phenomena: (1) water evaporation at the product surface, (2) vapor transport within the surrounding ambient air, and (3) water diffusion inside the solid porous matrix from its core towards the surface. Thus, when the external heat and mass transfers do not limit the whole operation through adequate airflow temperature, relative humidity, and velocity (NER), only internal transfers may intervene as limiting processes. 

To address the mass transfer limiting process, it is necessary to increase the effective exchange surface area and the porosity of the food material; for that, this study evaluated the effect of the swell-drying operation. Swell drying is an innovative drying operation that consists of coupling airflow drying with DIC texturing. In this study, the effect of swell drying on mango drying kinetics was evaluated through the starting accessibility (*δW*), the apparent drying coefficient (*D_app_*), and the time to obtain a final moisture content of 20% d.b (t_f_ = 20% d.b). Results showed that both the treatment time and the steam pressure impacted the *D_app_* and the *δW*. By comparing to the control (only CAD), SD (0.54 MPa and 48 s) increased to 12.2 and 2.7 times the *D_app_* and the *δW*, respectively. Moreover, SD triggers a significant reduction in post-drying time (tf = 20% d.b); this being 2.4 h vs. 30.8 h. These results could be linked to the expansion of the internal pores of mangoes generated by the instant autovaporization of residual water triggered by DIC treatment.

Swell drying has a significant positive effect on the drying kinetics of mangoes, by increasing water diffusivity, increasing the starting accessibility, reducing the drying time, and by remedying the shrinkage and collapse of dried products.

## Figures and Tables

**Table 1:** Applied experimental design of selected DIC processing parameters.

DIC Trial	Steam Pressure (MPa)	Thermal Treatment Time (s)
1	0.4	30
2	0.6	30
3	0.4	55
4	0.54	48
5	0.54	12
6	0.4	30
7	0.26	12
8	0.26	48
9	0.4	30
10	0.2	30
11	0.4	5

**Table 2:** Experimental data of drying kinetics of mangoes. Evolution of moisture content in dry basis (g H_2_O/g dry matter) in the function of the time *W = f(t)*.

Time (Min)	Sample											
	RM	DIC 1	DIC 2	DIC 3	DIC 4	DIC 5	DIC 6	DIC 7	DIC 8	DIC 9	DIC 10	DIC 11
0	0.38	0.34	0.38	0.37	0.39	0.38	0.36	0.34	0.42	0.36	0.35	0.4
1	0.37	0.33	0.37	0.36	0.38	0.37	0.35	0.33	0.41	0.34	0.33	0.39
5	0.36	0.32	0.35	0.34	0.36	0.36	0.33	0.32	0.4	0.33	0.32	0.38
15	0.35	0.3	0.32	0.32	0.33	0.33	0.31	0.3	0.37	0.3	0.3	0.36
30	0.34	0.27	0.3	0.29	0.3	0.3	0.29	0.28	0.35	0.28	0.28	0.33
60	0.33	0.24	0.25	0.25	0.25	0.26	0.24	0.25	0.31	0.24	0.24	0.3
90	0.32	0.21	0.23	0.23	0.22	0.24	0.22	0.23	0.28	0.22	0.22	0.28
120	0.31	0.2	0.21	0.21	0.21	0.22	0.21	0.21	0.27	0.2	0.2	0.27
180	0.3	0.17	0.17	0.18	0.18	0.19	0.18	0.19	0.23	0.17	0.17	0.24
240	0.29	0.16	0.16	0.16	0.17	0.18	0.16	0.18	0.22	0.15	0.16	0.23
300	0.29	0.15	0.15	0.15	0.16	0.16	0.15	0.17	0.21	0.14	0.15	0.22
360	0.28	0.14	0.14	0.14	0.15	0.15	0.14	0.16	0.2	0.13	0.14	0.21
1320	0.24	0.09	0.11	0.11	0.13	0.12	0.11	0.11	0.15	0.09	0.1	0.16

**Table 3:** Experimental data and predicted model results of the apparent drying coefficient of mangoes.

Sample	Steam Pressure (MPa)	Time (s)	Experimental Apparent Drying Coefficient (10^−10^ m^2^/s)	Predicted Model Apparent Drying Coefficient (10^−10^ m^2^/s)
RM	NA	NA	1.5514	NA
DIC 1	0.4	30	11.1469	12.2733
DIC 2	0.6	30	16.6039	18.2010
DIC 3	0.4	55	14.7236	14.4522
DIC 4	0.54	48	18.9215	18.2091
DIC 5	0.54	12	13.2968	11.7805
DIC 6	0.4	30	11.9720	12.2733
DIC 7	0.26	12	5.8954	6.0536
DIC 8	0.26	48	11.1147	12.0727
DIC 9	0.4	30	13.7009	12.2733
DIC 10	0.2	30	10.7492	9.8124
DIC 11	0.4	5	4.7879	5.8080

**Table 4:** Analysis of variance for apparent drying coefficient-MANGO.

Source	Sum of Squares	DDL	Mean Square	Ratio F	Probability
A:Pressure	68.9607	1	68.9607	30.29	0.0027
B:Time	77.4713	1	77.4713	34.03	0.0021
AA	4.07404	1	4.07404	1.79	0.2386
AB	0.0410873	1	0.0410873	0.02	0.8984
BB	6.97053	1	6.97053	3.06	0.1405

**Table 5:** Experimental data and predicted model results of the starting accessibility of mangoes.

Sample	Steam Pressure (MPa)	Time (s)	Experimental Starting Accessibility (g H_2_O/g Dry Matter)	Predicted Model Starting Accessibility (g H_2_O/g Dry Matter)
RM	NA	NA	0.0334	NA
DIC 1	0.4	30	0.0692	0.0741
DIC 2	0.6	30	0.0878	0.0889
DIC 3	0.4	55	0.0784	0.0809
DIC 4	0.54	48	0.0904	0.0882
DIC 5	0.54	12	0.0763	0.0773
DIC 6	0.4	30	0.0741	0.0741
DIC 7	0.26	12	0.0607	0.0622
DIC 8	0.26	48	0.0767	0.0751
DIC 9	0.4	30	0.079	0.0741
DIC 10	0.2	30	0.0691	0.0690
DIC 11	0.4	5	0.0659	0.0643

**Table 6:** Analysis of variance for Starting_accessibility-MANGO.

Source	Sum of Squares	DDL	Mean Square	Ratio F	Probability
A:Pressure	0.000388449	1	0.000388449	27.99	0.0032
B:Time	0.000285338	1	0.000285338	20.56	0.0062
AA	0.000031352	1	0.000031352	2.26	0.1931
AB	9.03 × 10^−07^	1	9.03 × 10^−07^	0.07	0.8089
BB	3.55781 × 10^−06^	1	3.55781 × 10^−06^	0.26	0.6341

**Table 7:** Experimental data and predicted model results of Time for obtaining a final moisture content of 20% d.b.

Sample	Steam Pressure (MPa)	Time (s)	Experimental Time for Obtaining Final Moisture of 0.2 g H_2_O/g Dry Matter (Min)	Predicted Model Time for Obtaining Final Moisture of 0.2 g H_2_O/g Dry Matter (Min)
RM	NA	NA	1819	NA
DIC 1	0.4	30	168	159
DIC 2	0.6	30	158	142
DIC 3	0.4	55	156	201
DIC 4	0.54	48	170	147
DIC 5	0.54	12	183	222
DIC 6	0.4	30	166	159
DIC 7	0.26	12	220	234
DIC 8	0.26	48	256	209
DIC 9	0.4	30	144	159
DIC 10	0.2	30	171	193
DIC 11	0.4	5	314	270

## Data Availability

Not applicable.

## Nomenclature

δW	Starting accessibility (g H2O/g dry matter)
$D_{eff}$	Effective W water content (kg water/kg dry matter) in the solid matrix
$D_{app}$	Apparent drying coefficient (m^2^ s^−1^)
$\rho_{d}$	apparent density of dry material kg#xB7;m^−3^
$\rho_{w}$	apparent density of water in the material kg·m^−3^
$\nu_{w}$	the absolute velocity of water within the material (ms^−1^)
$\nu_{d}$	the absolute velocity of the dry material (ms^−1^)
***W***	water content (kg water/kg dry matter) in the solid matrix
***W*_1_**	water content at the starting diffusion time
***W*_0_**	value of moisture content calculated from diffusion model extrapolated to t = 0 (% db)
***W_∞_***	equilibrium water content at a very long time t→∞ (kg water/kg dry matter)
***W_i_***	initial water content (kg water/kg dry matter)
